# Supplementary material for: A Likelihood Approach for Real-Time Calibration of Stochastic Compartmental Epidemic Models
Source: PLoS Comput Biol. 2017 Jan 17;13(1):e1005257. doi: 10.1371/journal.pcbi.1005257 (PMC5240920; doi:10.1371/journal.pcbi.1005257)
Supplement: S1 File — (TAR.GZ) [file pcbi.1005257.s014.tar.gz › HSPH_Online-SI-Revision/PF08/n10-mild/n10-mild_PF2_table0.pdf]

1	newly 3 weeks spec	176.	285.08	{254., 310.}	61.9773	0.208292	newly 3 weeks cumul	530.	866.82	{803., 924.}	63.5509	0.213073	newly 5 weeks spec	135.	262.61	{232., 288.}	94.5259	0.287491	newly 5 weeks cumul	825.	1405.22	{1314., 1481.}	70.3297	0.230746	newly 50 weeks cumul	2160.	3797.5	{3578., 4017.}	75.8102	0.244649
2	newly 3 weeks spec	316.	525.54	{426., 594.}	66.3481	0.217867	newly 3 weeks cumul	1222.	1562.76	{1292., 1776.}	28.4108	0.106312	newly 5 weeks spec	183.	472.43	{399., 533.}	158.158	0.408924	newly 5 weeks cumul	1613.	2539.03	{2118., 2850.}	57.4104	0.194334	newly 55 weeks cumul	2172.	5423.25	{4522., 5881.}	149.689	0.395232
3	newly 3 weeks spec	434.	596.13	{481., 726.}	38.1452	0.135864	newly 3 weeks cumul	1373.	1738.7	{1427., 2104.}	27.8412	0.103305	newly 5 weeks spec	309.	534.55	{394., 638.}	73.0129	0.231486	newly 5 weeks cumul	2076.	2852.36	{2299., 3438.}	37.894	0.135299	newly 58 weeks cumul	2924.	5800.76	{4232., 6708.}	98.3844	0.291864
4	newly 3 weeks spec	417.	400.26	{316., 497.}	12.494	0.0568907	newly 3 weeks cumul	1378.	1208.09	{947., 1513.}	15.516	0.0756757	newly 5 weeks spec	196.	364.49	{291., 425.}	85.9643	0.26475	newly 5 weeks cumul	1896.	1957.72	{1575., 2378.}	12.9388	0.0547971	newly 53 weeks cumul	2527.	4663.9	{4166., 5124.}	84.5627	0.263996
5	newly 3 weeks spec	111.	59.25	{48., 70.}	46.6216	0.277	newly 3 weeks cumul	344.	201.47	{186., 217.}	41.4331	0.232983	newly 5 weeks spec	94.	44.93	{35., 54.}	52.2021	0.329055	newly 5 weeks cumul	547.	297.6	{281., 319.}	45.5941	0.265008	newly 33 weeks cumul	2021.	589.68	{516., 662.}	70.8224	0.5371
6	newly 3 weeks spec	331.	510.62	{354., 699.}	55.1057	0.180218	newly 3 weeks cumul	1004.	1422.49	{983., 1893.}	43.5468	0.148637	newly 5 weeks spec	169.	504.94	{334., 673.}	198.781	0.462561	newly 5 weeks cumul	1390.	2445.32	{1683., 3207.}	76.0144	0.234353	newly 59 weeks cumul	2050.	5743.09	{4288., 7081.}	180.151	0.439722
7	newly 3 weeks spec	277.	408.32	{331., 496.}	47.4079	0.162868	newly 3 weeks cumul	873.	1194.08	{974., 1479.}	36.9485	0.13135	newly 5 weeks spec	186.	388.13	{313., 465.}	108.672	0.313375	newly 5 weeks cumul	1277.	1981.29	{1604., 2413.}	55.1519	0.185723	newly 55 weeks cumul	1892.	5003.65	{4022., 5662.}	164.464	0.418411
8	newly 3 weeks spec	269.	291.51	{264., 325.}	9.86989	0.0402694	newly 3 weeks cumul	743.	888.16	{831., 963.}	19.537	0.0767949	newly 5 weeks spec	224.	262.57	{232., 289.}	17.433	0.0682875	newly 5 weeks cumul	1216.	1430.69	{1327., 1535.}	17.6554	0.0699626	newly 50 weeks cumul	3026.	3713.31	{3463., 3925.}	22.9442	0.0893086
9	newly 3 weeks spec	476.	399.11	{334., 459.}	16.6996	0.0824935	newly 3 weeks cumul	1520.	1181.93	{1015., 1369.}	22.2414	0.112408	newly 5 weeks spec	288.	374.5	{309., 430.}	30.1319	0.11123	newly 5 weeks cumul	2165.	1943.54	{1659., 2201.}	11.6702	0.0557137	newly 54 weeks cumul	2749.	4865.3	{3978., 5401.}	76.9844	0.245343
10	newly 3 weeks spec	159.	308.98	{279., 350.}	94.327	0.286566	newly 3 weeks cumul	644.	927.61	{867., 998.}	44.0388	0.157432	newly 5 weeks spec	135.	284.82	{257., 315.}	110.978	0.322195	newly 5 weeks cumul	960.	1509.62	{1402., 1608.}	57.2521	0.195632	newly 51 weeks cumul	1852.	4026.63	{3771., 4325.}	117.421	0.336421
11	newly 3 weeks spec	170.	356.53	{236., 458.}	109.724	0.310492	newly 3 weeks cumul	592.	1027.7	{745., 1304.}	73.598	0.230135	newly 5 weeks spec	147.	352.04	{242., 461.}	139.483	0.367528	newly 5 weeks cumul	872.	1735.91	{1246., 2204.}	99.0722	0.289432	newly 56 weeks cumul	1526.	4861.27	{3914., 5806.}	218.563	0.497372
12	newly 3 weeks spec	240.	830.69	{703., 947.}	246.121	0.536331	newly 3 weeks cumul	1103.	2529.87	{2081., 3026.}	129.363	0.357403	newly 5 weeks spec	100.	656.66	{563., 732.}	556.66	0.814758	newly 5 weeks cumul	1384.	3941.92	{3325., 4575.}	184.821	0.452146	newly 58 weeks cumul	1560.	6658.81	{5472., 7271.}	326.847	0.627678
13	newly 3 weeks spec	357.	315.26	{282., 366.}	13.1148	0.0623686	newly 3 weeks cumul	1370.	970.38	{854., 1098.}	29.1693	0.151752	newly 5 weeks spec	224.	280.51	{241., 315.}	25.2545	0.0957578	newly 5 weeks cumul	1865.	1551.3	{1378., 1749.}	16.8311	0.0817493	newly 50 weeks cumul	2415.	3809.97	{3543., 4084.}	57.7627	0.197265
14	newly 3 weeks spec	307.	333.95	{295., 375.}	10.7524	0.0435803	newly 3 weeks cumul	1011.	1006.9	{913., 1124.}	6.37191	0.0277697	newly 5 weeks spec	225.	302.36	{271., 334.}	34.3822	0.126799	newly 5 weeks cumul	1501.	1631.56	{1483., 1779.}	9.62025	0.039282	newly 51 weeks cumul	2614.	4151.85	{3864., 4428.}	58.8313	0.200246
15	newly 3 weeks spec	226.	311.68	{277., 353.}	37.9115	0.13732	newly 3 weeks cumul	696.	907.47	{809., 1014.}	30.3836	0.113366	newly 5 weeks spec	226.	304.48	{269., 337.}	34.7257	0.12721	newly 5 weeks cumul	1140.	1526.28	{1362., 1695.}	33.8842	0.125046	newly 53 weeks cumul	3240.	4609.56	{4359., 4890.}	42.2704	0.152546
16	newly 3 weeks spec	319.	434.44	{310., 522.}	37.8558	0.134776	newly 3 weeks cumul	1150.	1276.91	{940., 1561.}	17.9522	0.0726262	newly 5 weeks spec	224.	411.94	{297., 489.}	83.9018	0.257336	newly 5 weeks cumul	1670.	2118.07	{1553., 2562.}	29.8425	0.11086	newly 54 weeks cumul	2574.	5093.08	{3888., 5795.}	97.8664	0.291052
17	newly 3 weeks spec	105.	285.58	{260., 310.}	171.981	0.433467	newly 3 weeks cumul	312.	849.36	{800., 896.}	172.231	0.434491	newly 5 weeks spec	82.	275.39	{250., 304.}	235.841	0.524879	newly 5 weeks cumul	477.	1407.3	{1321., 1501.}	195.031	0.469425	newly 51 weeks cumul	3040.	4119.9	{3922., 4302.}	35.523	0.131712
18	newly 3 weeks spec	181.	302.46	{269., 336.}	67.105	0.221527	newly 3 weeks cumul	580.	897.38	{813., 965.}	54.7207	0.188677	newly 5 weeks spec	194.	286.74	{262., 312.}	47.8041	0.168506	newly 5 weeks cumul	959.	1480.15	{1359., 1581.}	54.3431	0.187796	newly 51 weeks cumul	1686.	4158.52	{3976., 4357.}	146.65	0.391786
19	newly 3 weeks spec	266.	429.93	{354., 520.}	61.6278	0.203601	newly 3 weeks cumul	957.	1338.49	{1113., 1618.}	39.8631	0.141589	newly 5 weeks spec	162.	374.3	{301., 461.}	131.049	0.357106	newly 5 weeks cumul	1331.	2117.11	{1751., 2573.}	59.0616	0.197226	newly 54 weeks cumul	1835.	4401.86	{3477., 5406.}	139.883	0.372794
20	newly 3 weeks spec	319.	404.42	{290., 491.}	29.9749	0.110391	newly 3 weeks cumul	1048.	1205.03	{906., 1460.}	20.1823	0.0787503	newly 5 weeks spec	244.	372.79	{267., 470.}	53.6598	0.17881	newly 5 weeks cumul	1558.	1968.87	{1483., 2397.}	28.6772	0.105746	newly 55 weeks cumul	2688.	4587.92	{3316., 5560.}	70.8371	0.224704
21	newly 3 weeks spec	299.	499.38	{412., 567.}	67.0167	0.21932	newly 3 weeks cumul	1149.	1480.85	{1200., 1700.}	29.4056	0.109264	newly 5 weeks spec	209.	456.65	{377., 521.}	118.493	0.335784	newly 5 weeks cumul	1607.	2417.74	{1989., 2730.}	50.4505	0.174397	newly 55 weeks cumul	2136.	5335.9	{4392., 5887.}	149.808	0.394304
22	newly 3 weeks spec	242.	305.77	{265., 350.}	26.3512	0.0994255	newly 3 weeks cumul	748.	924.04	{824., 1057.}	23.5348	0.0898944	newly 5 weeks spec	169.	283.98	{239., 324.}	68.0355	0.222788	newly 5 weeks cumul	1116.	1506.09	{1359., 1698.}	34.9543	0.128477	newly 51 weeks cumul	2163.	4029.97	{3769., 4333.}	86.3139	0.269497
23	newly 3 weeks spec	259.	535.79	{451., 608.}	106.869	0.312421	newly 3 weeks cumul	963.	1563.7	{1331., 1794.}	62.378	0.207429	newly 5 weeks spec	207.	492.48	{404., 570.}	137.913	0.372473	newly 5 weeks cumul	1381.	2577.02	{2189., 2930.}	86.6054	0.267841	newly 56 weeks cumul	1616.	5655.43	{4655., 6254.}	249.965	0.541318
24	newly 3 weeks spec	173.	325.49	{288., 363.}	88.1445	0.272612	newly 3 weeks cumul	546.	967.19	{877., 1085.}	77.141	0.246928	newly 5 weeks spec	105.	306.1	{264., 353.}	191.524	0.462007	newly 5 weeks cumul	774.	1595.02	{1439., 1771.}	106.075	0.312701	newly 52 weeks cumul	1235.	4387.04	{3860., 4798.}	255.226	0.548755
25	newly 3 weeks spec	260.	453.96	{310., 609.}	74.8	0.231367	newly 3 weeks cumul	915.	1233.26	{902., 1641.}	37.1869	0.130869	newly 5 weeks spec	241.	477.99	{350., 612.}	98.3361	0.287584	newly 5 weeks cumul	1474.	2182.7	{1594., 2769.}	49.1194	0.16538	newly 59 weeks cumul	2031.	5980.24	{4731., 6842.}	194.448	0.463795
26	newly 3 weeks spec	226.	311.97	{276., 344.}	38.0398	0.138604	newly 3 weeks cumul	712.	950.08	{862., 1031.}	33.4382	0.124452	newly 5 weeks spec	102.	279.85	{253., 305.}	174.363	0.437007	newly 5 weeks cumul	971.	1529.75	{1412., 1652.}	57.5438	0.19671	newly 50 weeks cumul	1344.	3893.98	{3601., 4152.}	189.731	0.461443
27	newly 3 weeks spec	316.	378.91	{320., 429.}	20.9652	0.0805643	newly 3 weeks cumul	1146.	1147.26	{933., 1296.}	10.1571	0.044952	newly 5 weeks spec	192.	337.73	{284., 383.}	75.901	0.242169	newly 5 weeks cumul	1600.	1844.29	{1541., 2062.}	17.1944	0.0677342	newly 52 weeks cumul	1907.	4385.62	{4030., 4790.}	129.975	0.360196
28	newly 3 weeks spec	99.	35.64	{27., 43.}	64.	0.450607	newly 3 weeks cumul	324.	121.06	{108., 135.}	62.6358	0.429139	newly 5 weeks spec	91.	26.88	{21., 34.}	70.4615	0.53833	newly 5 weeks cumul	522.	178.17	{162., 196.}	65.8678	0.468053	newly 30 weeks cumul	1626.	355.2	{304., 416.}	78.155	0.663857
29	newly 3 weeks spec	301.	375.32	{312., 451.}	25.608	0.0958103	newly 3 weeks cumul	953.	1133.47	{962., 1341.}	19.7828	0.0756716	newly 5 weeks spec	234.	336.37	{271., 398.}	44.0726	0.154364	newly 5 weeks cumul	1506.	1829.01	{1555., 2153.}	21.9741	0.0834709	newly 53 weeks cumul	2280.	4358.75	{3314., 4967.}	91.1732	0.275758
30	newly 3 weeks spec	342.	371.3	{296., 448.}	15.0585	0.0611233	newly 3 weeks cumul	1048.	1132.67	{910., 1336.}	14.8464	0.0604404	newly 5 weeks spec	206.	335.5	{273., 398.}	62.8641	0.206584	newly 5 weeks cumul	1528.	1820.58	{1488., 2143.}	20.3586	0.0772123	newly 52 weeks cumul	2105.	4360.49	{3537., 4975.}	107.149	0.312593
31	newly 3 weeks spec	270.	464.9	{317., 591.}	72.3704	0.225002	newly 3 weeks cumul	910.	1273.25	{920., 1595.}	41.3154	0.141905	newly 5 weeks spec	207.	483.7	{347., 636.}	133.671	0.358723	newly 5 weeks cumul	1305.	2239.25	{1590., 2852.}	71.6682	0.225008	newly 59 weeks cumul	1766.	5986.32	{4883., 6940.}	238.976	0.525877
32	newly 3 weeks spec	121.	51.94	{40., 64.}	57.0744	0.373464	newly 3 weeks cumul	377.	181.72	{159., 202.}	51.7984	0.318635	newly 5 weeks spec	116.	41.01	{32., 50.}	64.6466	0.4589	newly 5 weeks cumul	611.	270.17	{242., 303.}	55.7823	0.356095	newly 32 weeks cumul	2466.	521.4	{438., 586.}	78.8564	0.677867
33	newly 3 weeks spec	364.	470.63	{401., 543.}	29.4258	0.109345	newly 3 weeks cumul	1353.	1407.73	{1207., 1641.}	9.31486	0.039113	newly 5 weeks spec	266.	421.71	{359., 491.}	58.5376	0.197011	newly 5 weeks cumul	1933.	2281.	{1939., 2648.}	18.6891	0.0725908	newly 53 weeks cumul	2442.	5107.09	{4745., 5605.}	109.136	0.318863
34	newly 3 weeks spec	262.	348.63	{296., 408.}	33.1336	0.120917	newly 3 weeks cumul	877.	1025.64	{865., 1195.}	18.1095	0.0700652	newly 5 weeks spec	247.	326.67	{271., 376.}	32.3279	0.118687	newly 5 weeks cumul	1378.	1691.4	{1430., 1961.}	22.8316	0.0867788	newly 53 weeks cumul	2645.	4538.1	{4134., 5038.}	71.5728	0.232896
35	newly 3 weeks spec	285.	430.79	{335., 520.}	51.1614	0.173738	newly 3 weeks cumul	970.	1322.68	{1023., 1542.}	36.4557	0.130337	newly 5 weeks spec	184.	367.82	{281., 435.}	99.9022	0.294615	newly 5 weeks cumul	1379.	2091.44	{1642., 2436.}	51.6635	0.176053	newly 53 weeks cumul	2032.	4445.48	{3605., 5093.}	118.774	0.336043
36	newly 3 weeks spec	365.	521.31	{430., 596.}	42.8247	0.151324	newly 3 weeks cumul	1132.	1571.75	{1278., 1790.}	38.8472	0.139231	newly 5 weeks spec	206.	459.81	{381., 530.}	123.209	0.34552	newly 5 weeks cumul	1604.	2526.77	{2043., 2822.}	57.5293	0.194271	newly 55 weeks cumul	2018.	5257.65	{4306., 5835.}	160.538	0.413097
37	newly 3 weeks spec	233.	524.83	{399., 651.}	125.249	0.344416	newly 3 weeks cumul	842.	1483.64	{1142., 1808.}	76.7435	0.241183	newly 5 weeks spec	124.	507.18	{376., 625.}	309.016	0.60467	newly 5 weeks cumul	1099.	2517.23	{1977., 3098.}	129.047	0.352978	newly 58 weeks cumul	1455.	5787.77	{4243., 6792.}	297.785	0.594741
38	newly 3 weeks spec	329.	403.62	{328., 477.}	23.8845	0.0897744	newly 3 weeks cumul	996.	1188.42	{992., 1413.}	21.012	0.0799946	newly 5 weeks spec	222.	371.6	{303., 444.}	67.3874	0.218863	newly 5 weeks cumul	1520.	1951.06	{1625., 2318.}	28.675	0.105604	newly 54 weeks cumul	2847.	4857.75	{4068., 5380.}	70.627	0.229542
39	newly 3 weeks spec	207.	538.33	{476., 598.}	160.063	0.413204	newly 3 weeks cumul	1062.	1589.91	{1413., 1749.}	49.709	0.173553	newly 5 weeks spec	83.	485.33	{423., 532.}	484.735	0.764698	newly 5 weeks cumul	1296.	2599.49	{2333., 2823.}	100.578	0.300707	newly 55 weeks cumul	1395.	5546.65	{4792., 6038.}	297.609	0.597552
40	newly 3 weeks spec	80.	29.97	{23., 37.}	62.5375	0.43358	newly 3 weeks cumul	250.	102.16	{92., 114.}	59.136	0.390169	newly 5 weeks spec	81.	21.55	{15., 29.}	73.3951	0.589891	newly 5 weeks cumul	408.	148.46	{134., 162.}	63.6127	0.440312	newly 28 weeks cumul	1434.	286.73	{232., 348.}	80.0049	0.703518
41	newly 3 weeks spec	318.	839.62	{680., 968.}	164.031	0.417752	newly 3 weeks cumul	1244.	2430.78	{1943., 2731.}	95.4003	0.287143	newly 5 weeks spec	73.	711.59	{553., 819.}	874.781	0.984012	newly 5 weeks cumul	1452.	3939.34	{3169., 4455.}	171.304	0.429951	newly 59 weeks cumul	1481.	7006.58	{5405., 7882.}	373.098	0.670814
42	newly 3 weeks spec	264.	301.72	{266., 337.}	15.053	0.0592024	newly 3 weeks cumul	803.	900.19	{801., 998.}	12.726	0.0508972	newly 5 weeks spec	207.	283.91	{247., 319.}	37.2415	0.135327	newly 5 weeks cumul	1233.	1482.	{1338., 1622.}	20.1946	0.0784966	newly 51 weeks cumul	1934.	4142.49	{3798., 4514.}	114.193	0.329276
43	newly 3 weeks spec	267.	670.	{562., 777.}	150.936	0.396279	newly 3 weeks cumul	1014.	2017.31	{1615., 2374.}	98.9458	0.295242	newly 5 weeks spec	120.	567.09	{491., 632.}	372.575	0.671331	newly 5 weeks cumul	1409.	3215.05	{2704., 3673.}	128.18	0.355445	newly 57 weeks cumul	1476.	6033.32	{5524., 6591.}	308.762	0.609074
44	newly 3 weeks spec	266.	332.96	{290., 378.}	25.2331	0.0956575	newly 3 weeks cumul	845.	981.35	{869., 1082.}	16.5266	0.0650335	newly 5 weeks spec	232.	313.35	{270., 352.}	35.0647	0.128147	newly 5 weeks cumul	1322.	1619.54	{1440., 1784.}	22.5401	0.0867226	newly 53 weeks cumul	2625.	4518.38	{4192., 4968.}	72.1288	0.234818
45	newly 3 weeks spec	490.	941.55	{837., 1026.}	92.1531	0.281826	newly 3 weeks cumul	1749.	2739.81	{2458., 2948.}	56.6501	0.1931	newly 5 weeks spec	208.	753.51	{660., 832.}	262.264	0.557216	newly 5 weeks cumul	2301.	4359.92	{3887., 4642.}	89.4794	0.275972	newly 60 weeks cumul	2545.	7250.9	{6608., 7803.}	184.908	0.453167
46	newly 3 weeks spec	320.	434.98	{311., 531.}	38.2875	0.135754	newly 3 weeks cumul	1046.	1251.89	{916., 1487.}	25.2361	0.0968735	newly 5 weeks spec	217.	426.5	{296., 514.}	96.5438	0.284535	newly 5 weeks cumul	1558.	2115.42	{1524., 2530.}	37.6714	0.133923	newly 57 weeks cumul	1999.	5275.18	{3810., 6333.}	163.891	0.414568
47	newly 3 weeks spec	449.	498.83	{374., 584.}	17.2806	0.0699722	newly 3 weeks cumul	1325.	1421.06	{1090., 1640.}	15.3072	0.0636559	newly 5 weeks spec	304.	482.19	{377., 553.}	58.6151	0.195563	newly 5 weeks cumul	2024.	2398.29	{1831., 2751.}	21.9837	0.0851628	newly 57 weeks cumul	2952.	5671.	{4454., 6281.}	92.107	0.280452
48	newly 3 weeks spec	220.	410.25	{320., 499.}	86.4773	0.264618	newly 3 weeks cumul	864.	1216.09	{971., 1503.}	41.0984	0.144189	newly 5 weeks spec	200.	381.54	{299., 459.}	90.77	0.27449	newly 5 weeks cumul	1267.	1997.65	{1611., 2423.}	57.6677	0.192342	newly 54 weeks cumul	1825.	4950.89	{4453., 5508.}	171.282	0.430467
49	newly 3 weeks spec	192.	306.1	{279., 335.}	59.4271	0.201447	newly 3 weeks cumul	594.	928.37	{883., 987.}	56.2912	0.193471	newly 5 weeks spec	164.	277.6	{248., 310.}	69.2683	0.22697	newly 5 weeks cumul	949.	1499.37	{1413., 1589.}	57.9947	0.198146	newly 50 weeks cumul	2640.	3943.97	{3701., 4154.}	49.3928	0.173708
50	newly 3 weeks spec	145.	316.55	{284., 348.}	118.31	0.337299	newly 3 weeks cumul	506.	967.39	{894., 1043.}	91.1838	0.28054	newly 5 weeks spec	93.	286.43	{256., 316.}	207.989	0.487092	newly 5 weeks cumul	715.	1557.14	{1444., 1666.}	117.782	0.337275	newly 50 weeks cumul	1140.	3965.27	{3670., 4218.}	247.831	0.540316
